# Polarization-Independent Broadband Infrared Selective Absorber Based on Multilayer Thin Film

**DOI:** 10.3390/nano15090678

**Published:** 2025-04-29

**Authors:** Shenglan Wu, Hao Huang, Xin Wang, Chunhui Tian, Zhenyong Huang, Zhiyong Zhong, Shuang Liu

**Affiliations:** 1 School of Optoelectronic Science and Engineering, University of Electronic Science and Technology of China, Chengdu 611731, China; 2 School of Electronic Engineering, Chengdu Technological University, Chengdu 611730, China; 3 School of Electronic Science and Engineering, University of Electronic Science and Technology of China, Chengdu 611731, China

**Keywords:** infrared absorber, selective absorption, localized surface plasmon resonance (LSPR), F-P cavity resonance, multilayers

## Abstract

Spectrally selective infrared absorbers play a pivotal role in enabling optoelectronic applications such as infrared detection, thermal imaging, and photothermal conversion. In this paper, a dual-band wide-spectrum infrared selective absorber based on a metal–dielectric multilayer structure is designed. Through optimized design, the absorptance of the absorber reaches the peak values of 0.87 and 1.0 in the target bands (3–5 μm and 8–14 μm), while maintaining a low absorptance of about 0.2 in the non-working bands of 5–8 μm, with excellent spectral selectivity. By analyzing the Poynting vector and loss distribution, the synergistic mechanism of the ultra-thin metal localized enhancement effect, impedance matching, and intrinsic absorption of the material is revealed. This structure exhibits good polarization-insensitive characteristics and angle robustness within a large incident angle range, showing strong adaptability to complex optical field environments. Moreover, the proposed planarized structure design is compatible with standard fabrication processes and has good scalability, which can be applied to other electromagnetic wave bands. This research provides new design ideas and technical solutions for advanced optoelectronic applications such as radiation cooling, infrared stealth, and thermal radiation regulation.

## 1. Introduction

The manipulation of electromagnetic wave absorption in specific spectral bands has become a cornerstone for advancing technologies in infrared detection [1,2], thermal photovoltaic systems [3,4,5,6], and infrared stealth [7,8,9]. Within the infrared spectrum, mid-wave infrared (MWIR, 3–5 μm) and long-wave infrared (LWIR, 8–14 μm) have attracted significant attention due to their unique physical properties and application values. The LWIR band, which coincides with the Earth’s atmospheric transmission window (8–14 μm), enables efficient thermal exchange with the cold universe in radiative cooling systems [10,11,12,13,14]. On the other hand, the MWIR band demonstrates high sensitivity to blackbody radiation from high-temperature objects and molecular vibrational transitions [15,16,17], playing a critical role in industrial temperature measurement, gas sensing [18,19], and military reconnaissance [20]. Achieving differential spectral responses in these two separated bands—namely, constructing efficient absorption channels in MWIR and LWIR regions while suppressing energy absorption in the intermediate band (5–8 μm)—is paramount for enhancing optoelectronic device performance. In infrared detection systems, such selective absorption can significantly reduce background thermal noise [21]; in radiative cooling applications, it optimizes the net radiative heat dissipation efficiency to the universe [22,23]. However, conventional material systems are constrained by their continuous spectral response characteristics, making it challenging to simultaneously realize absorption enhancement in two separated bands and effective suppression in the intermediate band within a single device. This technical bottleneck urgently demands breakthroughs through innovative design approaches.

In recent years, spectral tailoring techniques based on micro-nano structures have provided innovative approaches for modulating the infrared radiative properties. Metamaterials, through the design of subwavelength artificial structural units, utilize the synergistic effect between localized surface plasmon resonance (LSPR) and magnetic response to achieve near-perfect absorption in specific wavelength bands [24,25,26]. These structures typically consist of noble metal nanoparticles or antenna arrays, which exhibit excellent selective absorption properties in the mid-infrared (MIR) band by exciting collective oscillation modes of conduction electrons [27,28]. However, their fabrication relies on high-precision nanofabrication techniques such as electron beam lithography and reactive ion etching [29,30,31], leading to process complexity and challenges in achieving large-area application. Photonic crystals generate photonic bandgaps (PBGs) through periodically arranged dielectric structures, controlling electromagnetic wave propagation [32]. Nevertheless, the angular sensitivity of photonic crystals imposes significant limitations on practical applications—variations in incident angles can cause Bragg condition mismatches, resulting in bandgap position shifts [33]. Multilayer thin-film structures based on Fabry–Pérot (F-P) cavity resonance achieve spectral selective absorption through interference effects [34,35]. These structures are typically composed of alternating high/low refractive index materials, forming standing wave resonances in specific wavelength bands through optimized layer thickness and interfacial reflectivity design. Compared with metamaterials and photonic crystals, multilayer structures offer compatibility with mature deposition processes such as magnetron sputtering. However, they are constrained by the narrowband nature of cavity mode resonances, making them unsuitable for broadband modulation requirements.

In this work, a wide band infrared absorber based on metal–dielectric multilayer heterostructures is proposed to realize selective absorption regulation in 3–14 μm. Through in-depth analysis of each dielectric layer, the regulation rules of selective absorbing materials are revealed. Impedance matching enhances the spectral selection, the local enhancement effect of ultra-thin metal, F-P cavity resonance, enhances the local light field, and synergies with the intrinsic absorption of materials achieve high absorption. In addition, the design is remarkably robust in terms of polarization state and incident angle, and has strong adaptability to a complex light field. The structure has no in-plane pattern design, avoids lithography and submicron pattern etching, is compatible with large-scale manufacturing technology, which has good scalability, and provides a solid theoretical basis for the design and application of wideband infrared absorption materials.

## 2. Design and Method

**Figure 1:**
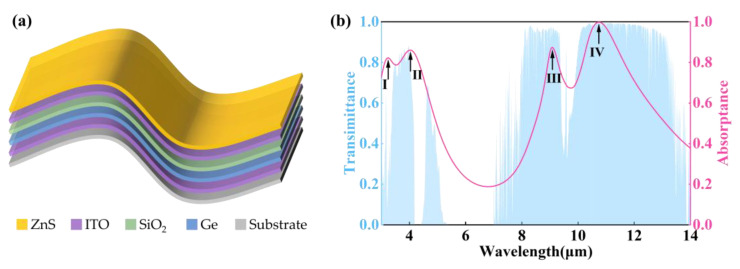
(**a**) The schematic diagram of MDDMD; (**b**) the atmospheric transmittance spectrum (blue area) and the absorptance spectrum (pink line) of the MDDMD.

To meet the demands of large-scale engineering applications, and address the limitations of traditional metal–dielectric–metal (MDM) structures, such as a narrow working bandwidth and full width at half maximum (FWHM), this study proposes a multilayer thin-film structure composed of metal–dielectric–dielectric–metal–dielectric (MDDMD) that constructs a gradient refractive index for precise manipulation of electromagnetic waves. The design scheme for achieving dual-band wide-spectrum infrared absorption is presented in Figure 1a. Considering the unique plasma resonance characteristics of indium tin oxide (ITO), it demonstrates pronounced metallic behavior in the infrared band [36,37]. Therefore, ITO is selected as a reflective and lossy metallic material [38]. To achieve and optimize selective absorption performance, the dielectric layer is the key part of the structure, and its material selection is very important. The core dielectric layer is composed of germanium (Ge), which exhibits exceptional transparency, a high refractive index (*n* ≈ 4.2), and negligible dispersion in the infrared band, thereby providing an ideal foundation for selective absorption. Additionally, silicon dioxide (SiO_2_) and zinc sulfide (ZnS) are incorporated as auxiliary dielectric layers. Silicon dioxide exhibits local dispersion within the target band, whereas ZnS is dispersionless across the entire spectrum. The coordinated action of these dielectric materials enables precise regulation of impedance matching, effectively achieving selective absorption and broadening the absorption bandwidth.

To optimize the performance of the multilayer thin-film absorber, we employed the wave optics and optimization modules of COMSOL Multiphysics 6.2 to analyze and iteratively optimize the parameters of the multilayers, ensuring the structure demonstrates optimal radiative selectivity within a specified wavelength range. To reduce the computational complexity and shorten the research period, 2D modeling is adopted with periodic boundary conditions applied to the side walls. It is assumed that there is a planar electromagnetic wave front at the two ports, and the excitation port is positioned at the boundary between the top perfectly matched layer (PML) and the air domain, whereas the corresponding transmission port is located at the interface between the bottom PML and the substrate. The optical properties (refractive index and extinction coefficients) of the dielectric materials are obtained from COMSOL’s internal materials database. The equivalent permittivity of ITO can be accurately described using the Drude model within this spectral range [39]:

(1) $$\varepsilon (\omega )=\varepsilon_{\infty }-\frac{\omega_{p}^{2}}{\omega (\omega +i\gamma )}$$

where *ε*_∞_ = 3.95 represents the relative permittivity at infinite frequency, *ω_p_* = 461 THz denotes the plasma frequency, and *γ* = 1.822 × 10^14^ Hz signifies the damping coefficient.

By adjusting the thickness of each dielectric layer, the phase distribution of the electromagnetic wave within the dielectric is modulated, enabling precise control over the spectral response of a specific band. During the optimization process, the thickness of the dielectric layers serves as the primary design variable. A merit function is formulated to quantify the degree of matching between the spectral performance and the target response, thereby selecting the optimal thickness parameter. The specific merit function is defined as follows:

(2) $$F(\lambda )=\frac{1}{L_{1}}\int_{3\;\mu m}^{5\;\mu m}{\left[1-A(\lambda )\right]}^{2}\cdot T_{atm}(\lambda )d\lambda +\frac{1}{L_{2}}\int_{5\;\mu m}^{8\;\mu m}A{\left(\lambda \right)}^{2}d\lambda +\frac{1}{L_{3}}\int_{8\;\mu m}^{14\;\mu m}{\left[1-A(\lambda )\right]}^{2}\cdot T_{atm}(\lambda )d\lambda$$

where *L_x_* _= 1,2,3_ is the length of each band interval, which is used to normalize the integral weight and avoid the optimization bias to the long band due to the difference in band width; *T_atm_*(*λ*) represents the atmospheric transmittance [blue area in Figure 1b] [40]; and *A*(*λ*) is the spectral emissivity of the multilayer thin film. The weight *T*_atm_ (λ) is used to ensure the maximization of absorption in the high-transmission area of the atmospheric window to meet the requirements of practical applications. Additionally, *A*(λ)^2^ is directly minimized to force the absorptance to approach zero, strictly suppress the absorption in 5–8 μm, and avoid the introduction of noise.

## 3. Results and Discussion

By systematically exploring the thickness combinations of the multilayer thin-film through parametric scanning, combining electromagnetic simulations to calculate the spectral response, and using the evaluation function to screen for the optimal thickness configuration, the optimized MDDMD multilayer thin-film structure exhibits a precisely engineered layer configuration with thicknesses of 300 nm (bottom), 620 nm, 260 nm, 10 nm, and 200 nm (top). Importantly, this structure demonstrates excellent compatibility with conventional thin-film deposition techniques, particularly magnetron sputtering, ensuring its scalability for large-area fabrication, which is a critical advantage for practical implementation. As demonstrated in Figure 1b, the structure manifests exceptional selective absorption characteristics, with its spectral response showing remarkable consistency with the target spectral response. In the 3–5 μm spectral range, the absorption spectrum fully overlaps with the atmospheric transmission window, and the peak absorptance reaches 0.86, which signifies stable broadband absorption performance. In the 8–14 μm band, the absorption characteristics are remarkable, where the peak absorption reaches 1 with a FWHM of 4.6 μm, indicating highly efficient broadband absorption. Notably, the structure also exhibits excellent out-of-band suppression characteristics in the non-target band (5–8 μm), with absorbance as low as 0.20, thereby providing a stark contrast to the target bands. This exceptional band selectivity effectively minimizes interference from non-target signals, manifesting significant practical implications for various applications.

**Figure 2:**
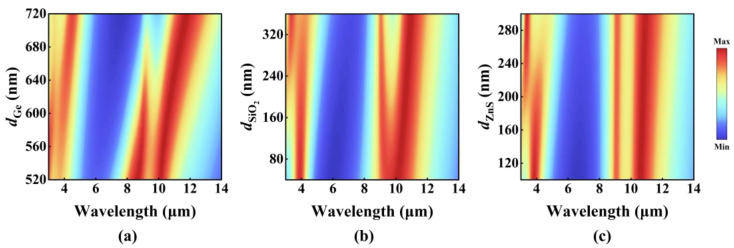
The spectrum of MDDMD varies with the thickness of the dielectric layer: (**a**) Ge; (**b**) SiO_2;_ (**c**) ZnS.

To further clarify the regulatory mechanism of each dielectric layer on the absorption characteristics, this study adopts the controlled variable method to systematically analyze the thickness of the Ge, SiO_2,_ and ZnS dielectric layers by parametric scanning (see Figure 2). As the core dielectric layer, the Ge layer’s thickness plays a critical role in modulating the selective absorption characteristic. An increase in the Ge layer thickness induces a notable redshift in the absorption peak, which can be attributed to the F-P resonance condition (*D* = m*λ*/2*n*, *D* is the cavity length, m = 1, 2, 3, …), providing a reasonable explanation for the observed phenomenon. The SiO_2_ layer exhibits unique spectral regulation characteristics within the 8–14 μm band: as thickness increases, the absorption peak progressively evolves from unimodal to bimodal configurations with expanding peak separation. This phenomenon arises from two underlying mechanisms: first, the resonant absorption peak experiences a redshift due to increased dielectric layer thickness; second, when the thickness increases, the intrinsic absorption effect of SiO_2_ decouples from the cavity resonance peak, leading to spectral splitting. The ZnS layer primarily influences the optical response in the 3–5 μm band, functioning as an anti-reflection coating to enhance impedance matching and adjust the position and width of the peak to promote absorption. Additionally, it serves as a protective layer to ensure structural stability. Through the cooperative interaction of these three dielectric layers, precise control over the spectral characteristics is achieved.

**Figure 3:**
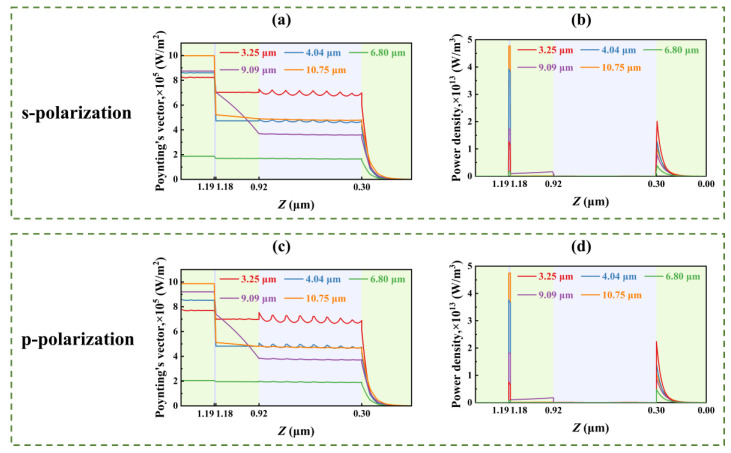
Poynting vectors and loss distributions along the vertical direction (i.e., *z*-axis) of the multilayer film at different wavelengths of 3.35 μm, 4.04 μm, 6.8 μm, 9.09 μm, and 10.75 μm: (**a**) Poynting vector (s-polarization); (**b**) electromagnetic loss (s-polarization); (**c**) Poynting vector (p-polarization); (**d**) electromagnetic loss (p-polarization). Note that the top surface of the film is at the left boundary.

In order to further investigate the selective infrared emission mechanism of the multilayer thin film, five characteristic wavelengths (3.25 μm, 4.04 μm, 6.8 μm, 9.09 μm, and 10.75 μm) were selected for subsequent research, corresponding to four absorption peaks [indicated by the arrow in Figure 1b] and the minimum value of 5–8 μm, respectively. Through the spatial distribution of the Poynting vector and loss perpendicular to the film plane (Figure 3), the transmission characteristics of radiation energy of different wavelengths in each layer are analyzed. The results indicate that the radiation energy exhibits significant fluctuating transmission characteristics due to the interference effects of the multilayer film structure and material properties.

At the wavelength of 3.25 μm, the Poynting vector demonstrates pronounced longitudinal penetration within the thin film structure, with approximately 80% of the incident electromagnetic wave energy transmitted to the bottom layer, which indicates that the absorption of this band is all from the loss of the bottom metal. At a wavelength of 9.09 μm, the radiation energy decays linearly within the SiO_2_ layer, exhibiting typical dielectric loss behavior. This phenomenon arises from the resonance of the Si-O-Si antisymmetric stretching mode (1080 cm^−1^), whose attenuation follows the Beer–Lambert law.

At wavelengths of 4.04 μm and 10.75 μm, the multi-layer film structures achieve 86% and 100% incident energy coupling efficiency, respectively, in which the energy attenuation of the metal layer accounts for over 93% of the total loss. This is mainly due to the synergistic effect of LSPR and F-P cavity resonance, which significantly enhance the light–matter interaction: the former forms a highly localized electromagnetic field on the metal surface by matching the frequency of incident light with the collective oscillation of free electrons in the metal nanostructure; and the latter extends the optical path through the optical interference effect between the multilayer films, and the two work together to enhance the optical field localization. Electron–lattice collision and metal ohmic loss constitute the main energy dissipation path: electrons collide inelastically with the lattice during oscillation, and the intrinsic resistance of the metal layer causes Joule heat.

In stark contrast to the aforementioned wavelengths, the 6.8 μm wavelength displays high reflection characteristics akin to those of metals. The energy entering the multilayer film is significantly lower than in other bands, with most of the energy being reflected, resulting in a reflectance exceeding 80%. These findings confirm that the multilayer structure achieves selective absorption in the 3–14 μm broadband through synergistic LSPR, F-P cavity resonance, phase modulation, and molecular vibration effects. Under p-polarization, the structure exhibits similar Poynting vector distributions [Figure 3c] and electromagnetic loss characteristics [Figure 3d] as under s-polarization, demonstrating its excellent polarization-independent properties.

**Figure 4:**
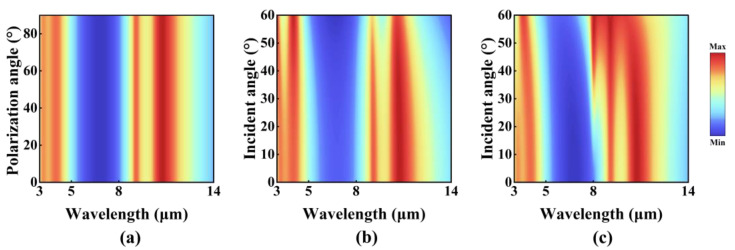
The absorption as a function of the polarization angle and incident angle for the proposed MDDMD: (**a**) polarization angle; (**b**) s-polarization incidence; (**c**) p-polarization incidence.

In practical engineering applications, the incident optical field frequently demonstrates complex characteristics, such as arbitrary polarization states and wide angular distributions. Consequently, the robustness of infrared-absorbing materials to the polarization direction and incident angle has become a critical technical indicator. Hence, this study systematically evaluates the sensitivity of the designed structure to polarization states and incident angles. Figure 4a depicts the absorption spectra of the structure under different polarization modes. As the polarization direction of the incident wave is continuously rotated from s-polarization to p-polarization, the absorption spectra show negligible shifts and amplitude attenuations. The absorption peak positions and amplitudes remain stable across the entire testing frequency range, indicating that the structure exhibits high insensitivity to incident light with arbitrary polarization states.

Moreover, the sensitivity of the structure to incident angles was analyzed, as illustrated in Figure 4b,c. Under s-polarization, when the incident angle increases from 0° to 60°, the absorption spectra exhibit tiny shifts (with a peak shift of approximately 0.1 μm), demonstrating excellent robustness to large incident angles. And under p-polarization, as the incident angle increases from 0° to 60°, the absorption peaks within the 8–14 μm atmospheric window experience a noticeable blue shift, accompanied by broadening of the FWHM. Nevertheless, the absorption peaks remain aligned with the atmospheric window, preserving selective absorption characteristics. Comparing Figure 4a–c, it can be clearly seen that the spectral response difference between s- and p-polarization gradually increases with the increase of incident angle. Despite the above differences, the designed structure demonstrates high tolerance to both polarization states and incident angles. This robustness substantially enhances its applicability in complex optical environments and provides a solid theoretical foundation for its practical implementation in multispectral optoelectronic devices.

## 4. Conclusions

In summary, a dual-band wide-spectrum infrared selective absorber based on a metal–dielectric multilayer structure is designed. Through the design of the gradient multilayer dielectric structure, it achieves broadband selective absorption in the 3–14 μm band. Firstly, through in-depth analysis of the dielectric layers, the necessity of using multilayer dielectric structures was verified. Further studies show that the synergistic mechanism of the localized enhancement effect of ultrathin metals, impedance matching, and intrinsic absorption of the materials is the key to achieving efficient absorption. After collaborative optimization, the maximum absorptance of this absorber reaches 0.86 in the 3–5 μm atmospheric window band and 1.0 in the 8–14 μm band, while the absorptance in the 5–8 μm band is as low as 0.2, demonstrating excellent selective absorption performance. Moreover, the absorber exhibits significant robustness in terms of polarization and incident angle, making it well suited to complex optical field conditions. This design optimizes the optical performance while considering the feasibility of large-scale production, laying a solid theoretical foundation for the engineering application of novel broadband infrared absorption materials. Moreover, it has good scalability and shows broad application prospects in infrared detection, thermal imaging, and infrared stealth fields.

## Data Availability

Data are contained within the article.
